# Supplementary material for: Predictive Bayesian selection of multistep Markov chains, applied to the detection of the hot hand and other statistical dependencies in free throws
Source: R Soc Open Sci. 2019 Mar 20;6(3):182174. doi: 10.1098/rsos.182174 (PMC6458367; doi:10.1098/rsos.182174)
Supplement: Supplementary Results [file rsos182174supp1.pdf]

A. Derivations

The marginal likelihood, also known as Bayes factor, is the expectation of the likelihood under the prior distribution. The logarithm of this quantity (LML) is

$$\begin{aligned}\mathrm{LML} &= \log \mathbb{E}_{\mathbf{p}}\left[\Pr(\mathbf{N} \mid \mathbf{p})\right] \\ &= \log \mathbb{E}_{\mathbf{p}}\left(\prod_{\mathbf{x}}\prod_{m=1}^{M} p_{\mathbf{x},m}^{N_{\mathbf{x},m}}\right) \\ &= \sum_{\mathbf{x}} \log\left(\frac{B(\mathbf{N}_{\mathbf{x}}+\alpha)}{B(\alpha)}\right). \qquad \text{(A 1)}\end{aligned}$$

The log predictive density (LPD) given a model defined by an inferred posterior distribution $\mathbf{p}|\mathbf{N}$ may be computed

$$\begin{aligned}\mathrm{LPD} &= \log \mathbb{E}_{\mathbf{p}|\mathbf{N}}\left[\Pr(\mathbf{N} \mid \mathbf{p})\right] \\ &= \log \mathbb{E}_{\mathbf{p}|\mathbf{N}}\left(\prod_{\mathbf{x}}\prod_{m=1}^{M} p_{\mathbf{x},m}^{N_{\mathbf{x},m}}\right) \\ &= \sum_{\mathbf{x}} \log\left(\frac{B(2\mathbf{N}_{\mathbf{x}}+\alpha)}{B(\mathbf{N}_{\mathbf{x}}+\alpha)}\right). \qquad \text{(A 2)}\end{aligned}$$

The log pointwise predictive density (LPPD), requires partition of data into disjoint “points.” Treating trajectories as points yields

$$\begin{aligned}\mathrm{LPPD} &= \sum_{j}\sum_{\mathbf{x}} \log \mathbb{E}_{\mathbf{p}_{\mathbf{x}}|\mathbf{N}_{\mathbf{x}}}\left[\Pr\left(\mathbf{N}_{\mathbf{x}}^{(j)} \mid \mathbf{p}_{\mathbf{x}}\right)\right] \\ &= \sum_{j}\sum_{\mathbf{x}} \log \mathbb{E}_{\mathbf{p}_{\mathbf{x}}|\mathbf{N}_{\mathbf{x}}}\left(\prod_{m=1}^{M} p_{\mathbf{x},m}^{N_{\mathbf{x},m}^{(j)}}\right) \\ &= \sum_{j}\sum_{\mathbf{x}} \log\left(\frac{B(\mathbf{N}_{\mathbf{x}}+\mathbf{N}_{\mathbf{x}}^{(j)}+\alpha)}{B(\mathbf{N}_{\mathbf{x}}+\alpha)}\right). \qquad \text{(A 3)}\end{aligned}$$

The LOO, as defined in this manuscript, is similar to the LPPD. For the LOO, each of the pointwise posterior distributions is computed after leaving out the corresponding trajectory. Hence,

$$\begin{aligned}\mathrm{LOO} &= -2\sum_{j}\sum_{\mathbf{x}} \log \mathbb{E}_{\mathbf{p}_{\mathbf{x}}|\mathbf{N}_{\mathbf{x}}\setminus\mathbf{N}_{\mathbf{x}}^{(j)}}\left[\Pr\left(\mathbf{N}_{\mathbf{x}}^{(j)} \mid \mathbf{p}_{\mathbf{x}}\right)\right] \\ &= -2\sum_{j}\sum_{\mathbf{x}} \log \mathbb{E}_{\mathbf{p}_{\mathbf{x}}|\mathbf{N}_{\mathbf{x}}\setminus\mathbf{N}_{\mathbf{x}}^{(j)}}\left(\prod_{m=1}^{M} p_{\mathbf{x},m}^{N_{\mathbf{x},m}^{(j)}}\right) \\ &= -2\sum_{j}\sum_{\mathbf{x}} \log\left(\frac{B(\mathbf{N}_{\mathbf{x}}-\mathbf{N}_{\mathbf{x}}^{(j)}+\mathbf{N}_{\mathbf{x}}^{(j)}+\alpha)}{B(\mathbf{N}_{\mathbf{x}}-\mathbf{N}_{\mathbf{x}}^{(j)}+\alpha)}\right). \qquad \text{(A 4)}\end{aligned}$$

For the WAIC, the two variants of complexity parameters are

$$
\begin{aligned}
k_{\mathrm{WAIC1}} &= 2\mathrm{LPPD} - 2\sum_j \sum_{\mathbf{x}} \mathbb{E}_{\mathbf{p_x}|\mathbf{N}} \left[\log \mathbf{p_x}^{\mathbf{N}_{\mathbf{x}}^{(j)}}\right] \\
&= 2\mathrm{LPPD} - \sum_j \sum_{\mathbf{x}} \sum_{m=1}^{M} N_{\mathbf{x},m}^{(j)} \mathbb{E}_{\mathbf{p_x}|\mathbf{N_x}} \left(\log p_{\mathbf{x},m}\right) \\
&= 2\mathrm{LPPD} - 2\sum_j \sum_{\mathbf{x}} \sum_{m=1}^{M} N_{\mathbf{x},m}^{(j)} \left[\psi(N_{\mathbf{x},m} + \alpha_m) - \psi\left(N_{\mathbf{x}} + \sum_m \alpha_m\right)\right] \\
&= 2\mathrm{LPPD} - 2\sum_{\mathbf{x}} \sum_{m=1}^{M} N_{\mathbf{x},m} \left[\psi(N_{\mathbf{x},m} + \alpha_m) - \psi\left(N_{\mathbf{x}} + \sum_m \alpha_m\right)\right],
\end{aligned}
\tag{A 5}
$$

and

$$
\begin{aligned}
k_{\mathrm{WAIC2}} &= \sum_j \sum_{\mathbf{x}} \mathrm{var}_{\mathbf{p_x}} \left[\log \Pr\left(\mathbf{N}_{\mathbf{x}}^{(j)} \mid \mathbf{p_x}\right)\right] \\
&= \sum_j \sum_{\mathbf{x}} \mathrm{var}_{\mathbf{p_x}} \left\{ \log\left(\prod_{m=1}^{M} p_{\mathbf{x},m}^{N_{\mathbf{x},m}^{(j)}}\right)\right\} \\
&= \sum_j \sum_{\mathbf{x}} \mathrm{var}_{\mathbf{p_x}} \left[\sum_{m=1}^{M} N_{\mathbf{x},m}^{(j)} \log p_{\mathbf{x},m}\right] \\
&= \sum_j \sum_{\mathbf{x}} \sum_{m=1}^{M} \sum_{n=1}^{M} N_{\mathbf{x},m}^{(j)} N_{\mathbf{x},n}^{(j)} \mathrm{cov}\left(\log p_{\mathbf{x},m}, \log p_{\mathbf{x},n}\right) \\
&= \sum_j \sum_{\mathbf{x}} \sum_{m=1}^{M} \sum_{n=1}^{M} N_{\mathbf{x},m}^{(j)} N_{\mathbf{x},n}^{(j)} \left[\psi'\left(\alpha_n + N_{\mathbf{x},n}\right)\delta_{nm} - \psi'\left(\sum_m \alpha_m + N_{\mathbf{x}}\right)\right] \\
&= \sum_j \sum_{\mathbf{x}} \left[\sum_{m=1}^{M} [N_{\mathbf{x},m}^{(j)}]^2 \psi'(\alpha_m + N_{\mathbf{x},m}) - [N_{\mathbf{x}}^{(j)}]^2 \psi'\left(\sum_m \alpha_m + N_{\mathbf{x}}\right)\right].
\end{aligned}
\tag{A 6}
$$

The commonly used Deviance Information Criterion (DIC)

$$
\mathrm{DIC} = -2\sum_{\mathbf{x}} \log p\left(\mathbf{N_x} \mid \mathbf{p_x} = \mathbb{E}_{\mathbf{p_x}|\mathbf{N_x}} \mathbf{p_x}\right) + 2k_{\mathrm{DIC}} \tag{A 7}
$$

also resembles the WAIC, consisting of two variants in the computation of model complexity,

$$
\begin{aligned}
k_{\mathrm{DIC1}} &= -2\left\{\sum_{\mathbf{x}} \sum_{m=1}^{M} N_{\mathbf{x},m} \log\left(\frac{N_{\mathbf{x},m} + \alpha_m}{N_{\mathbf{x}} + \sum_m \alpha_m}\right) - \sum_j \sum_{\mathbf{x}} \mathbb{E}_{\mathbf{p_x}|\mathbf{N}} \log \mathbf{p_x}^{\mathbf{N}_{\mathbf{x}}^{(j)}}\right\} \\
&= 2\left\{\sum_{\mathbf{x}} \sum_{m=1}^{M} N_{\mathbf{x},m} \log\left(\frac{N_{\mathbf{x},m} + \alpha_m}{N_{\mathbf{x}} + \sum_m \alpha_m}\right) - \sum_j \sum_{\mathbf{x}} \sum_{m=1}^{M} \mathbf{N}_{\mathbf{x},m}^{(j)} \left[\psi(\alpha_m + N_{\mathbf{x},m}) - \psi\left(\sum_m \alpha_m + N_{\mathbf{x}}\right)\right]\right\} \\
&= 2\left\{\sum_{\mathbf{x}} \sum_{m=1}^{M} N_{\mathbf{x},m} \log\left(\frac{N_{\mathbf{x},m} + \alpha_m}{N_{\mathbf{x}} + \sum_m \alpha_m}\right) - \sum_{\mathbf{x}} \sum_{m=1}^{M} N_{\mathbf{x},m} \left[\psi(\alpha_m + N_{\mathbf{x},m}) - \psi\left(\sum_m \alpha_m + N_{\mathbf{x}}\right)\right]\right\},
\end{aligned}
\tag{A 8}
$$

and $k_{\mathrm{DIC2}} = 2\mathrm{var}_{\mathbf{p}|\mathbf{N}}\left[\log \Pr\left(\mathbf{N} \mid \mathbf{p}\right)\right]$, which may be computed

$$
\begin{aligned}
k_{\mathrm{DIC2}} &= 2\mathrm{var}_{\mathbf{p}_\mathbf{x}}\left[\sum_{\mathbf{x}}\sum_{m} N_{\mathbf{x},m}\log p_{\mathbf{x},m}\right] \\
&= 2\sum_{\mathbf{x}}\mathrm{var}_{\mathbf{p}_\mathbf{x}}\left(\sum_{m} N_{\mathbf{x},m}\log p_{\mathbf{x},m}\right) \\
&= 2\sum_{\mathbf{x}}\sum_{m}\sum_{n} N_{x,m}N_{x,n}\mathrm{cov}(\log p_{\mathbf{x},m}, \log p_{\mathbf{x},n}) \\
&= 2\sum_{\mathbf{x}}\sum_{m}\sum_{n} N_{x,m}N_{x,n}\left[\psi'(\alpha_m + N_{\mathbf{x},m})\delta_{mn} - \psi'\left(\sum_{m}\alpha_m + N_{\mathbf{x}}\right)\right] \\
&= 2\sum_{\mathbf{x}}\left(\sum_{m} N_{\mathbf{x},m}^2\psi'(\alpha_m + N_{\mathbf{x},m}) - (N_{\mathbf{x}})^2\psi'\left(\sum_{m}\alpha_m + N_{\mathbf{x}}\right)\right),
\end{aligned}
\tag{A 9}
$$

where δ_{mn} refers to the Kronecker delta function.

B. Supplemental results

In Fig. 6, it is apparent that models where $h = 0$ and $h = 1$ have comparable predictive power. As a form of permutation test, we consider resamplings without replacement of James' 2016–2017 free throws where within each game the order of his shot outcomes are scrambled. The results here (Fig. S1) are similar to those found in Fig. 6, where the statistical power of the information criteria are evaluated using simulated data.

Fig. S2 presents a version of the same tests performed in the main manuscript (where an eight state system is used) on a four-state system. In comparing Fig. 2 to Fig. S2, one finds consistent results. Note that on average the simulated trajectory lengths are shorter in the four state system relative to the eight state system due to the fact that there are fewer interior states relative to the number of absorbing states.

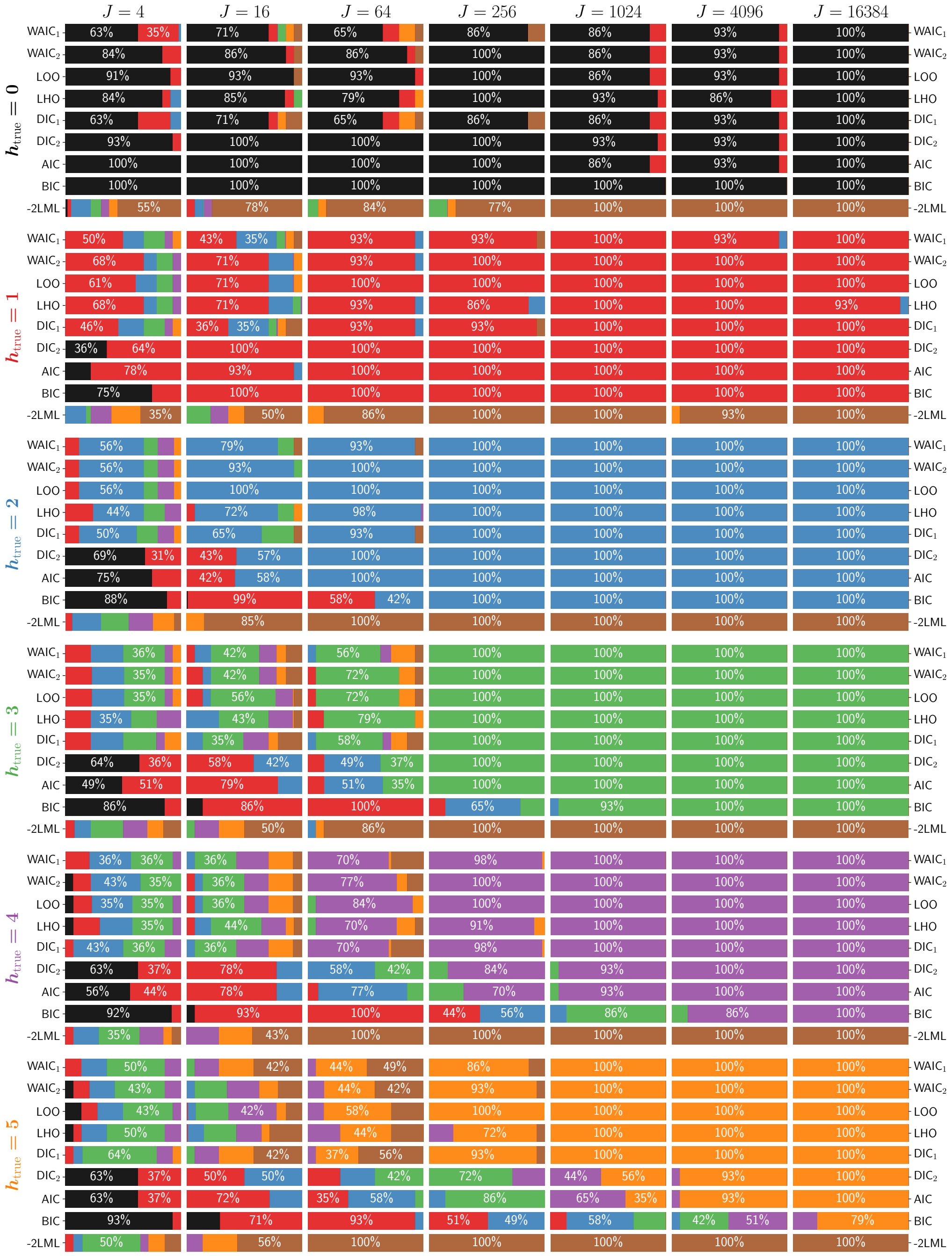

Figure S1. Permuting LeBron James' 16'–17' free throws. (a) Distributions of $\Delta\text{Criterion}(h) = \text{Criterion}(h) - \text{Criterion}(h=0)$ Evaluations of information criterion relative to $h = 0$ for resamplings without replacement of James' 16'-17' free throws. (b) Frequency of choosing $h = 0$: black, 1: red, 2: blue, 3: green.

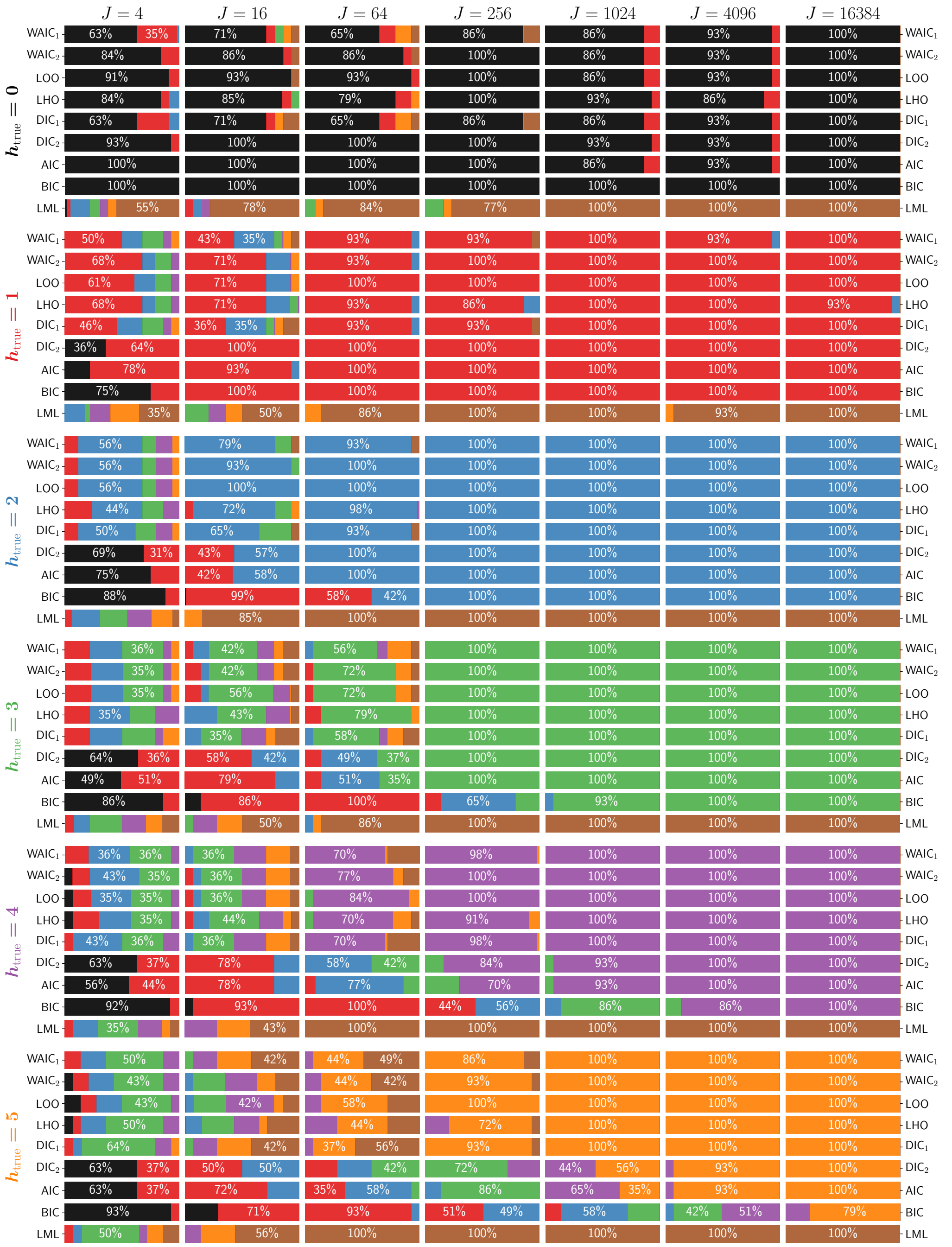

Figure S2. Chosen degree of memory h for $M=4$ system in simulations for varying true degrees of memory h_{true} and number of observed trajectories J. Choices made on basis of model with lowest value of given criterion. Rows correspond to model selection under a given degree of memory. Columns correspond to the number of trajectories. Depicted are the percent of simulations in which each degree of memory is selected using the different model evaluation criteria (percents of at least 20 are labeled). Colors coded based on degree of memory: (0: black, 1: red, 2: blue, 3: green, 4: purple, 5: orange). *Example:* For $h_{\text{true}}=1$ and $J=4$, the WAIC$_1$ criteria selected $h=1$ approximately 68% of the time.
